# “Enhanced acquisition of antibiotic-resistant intestinal *E. coli* during the first year of life assessed in a prospective cohort study”

**DOI:** 10.1186/s13756-019-0522-6

**Published:** 2019-05-20

**Authors:** Benjamin Hetzer, Dorothea Orth-Höller, Reinhard Würzner, Peter Kreidl, Michaela Lackner, Thomas Müller, Ludwig Knabl, Daniel Rudolf Geisler-Moroder, Alexander Mellmann, Özcan Sesli, Jeanett Holzknecht, Damia Noce, Orawan Boonpala, Noppadon Akarathum, Somporn Chotinaruemol, Martina Prelog, Peninnah Oberdorfer

**Affiliations:** 1 Department of Pediatrics I, Medical University of Innsbruck, Anichstraße 35, 6020 Innsbruck, Austria; 2 Division of Hygiene and Medical Microbiology, Medical University of Innsbruck, Schöpfstraße 41/III, 6020 Innsbruck, Austria; 3 Institute of Hygiene, University Hospital Münster, Robert-Koch-Strasse 41, 48149 Münster, Germany; 4 Center for Biomedicine, European Academy of Bolzano/Bozen (EURAC), Affiliated to the University of Lübeck, Via Galvani 31, 39100 Bolzano, Italy; 5 Division of Infectious Diseases, Department of Pediatrics, Faculty of Medicine, Chiang Mai University, Chiang Mai, Thailand; 6 Sanpathong Hospital, Ministry of Public Health, Chiang Mai, Thailand; 7 Department of Pediatrics, University Hospital Würzburg, Josef-Schneider-Strasse 2, 97080 Würzburg, Germany

**Keywords:** *Escherichia coli*, Antibiotic resistance, Multiresistance, Transmission, Persistence, Children, Neonates

## Abstract

**Background:**

Increasing bacterial resistance to antibiotics is a serious problem worldwide. We sought to record the acquisition of antibiotic-resistant *Escherichia coli* (*E. coli*) in healthy infants in Northern Thailand and investigated potential determinants.

**Methods:**

Stool samples from 142 infants after birth, at ages 2wk, 2mo, 4 to 6mo, and 1y, and parent stool samples were screened for *E. coli* resistance to tetracycline, ampicillin, co-trimoxazole, and cefazoline by culture, and isolates were further investigated for multiresistance by disc diffusion method. Pulsed-field gel electrophoresis was performed to identify persistent and transmitted strains. Genetic comparison of resistant and transmitted strains was done by multilocus sequence typing (MLST) and strains were further investigated for extra- and intra-intestinal virulence factors by multiplex PCR.

**Results:**

Forty-seven (33%) neonatal meconium samples contained resistant *E. coli.* Prevalence increased continuously: After 1y, resistance proportion (tetracycline 80%, ampicillin 72%, co-trimoxazole 66%, cefazoline 35%) almost matched those in parents. In 8 infants (6%), identical *E. coli* strains were found in at least 3 sampling time points (suggesting persistence). Transmission of resistant *E. coli* from parents to child was observed in only 8 families. MLST showed high diversity. We could not identify any virulence genes or factors associated with persistence, or transmission of resistant *E. coli*. Full-term, vaginal birth and birth in rural hospital were identified as risk factors for early childhood colonization with resistant *E. coli*.

**Conclusion:**

One third of healthy Thai neonates harboured antibiotic-resistant *E. coli* in meconium. The proportion of resistant *E. coli* increased during the first year of life almost reaching the value in adults. We hypothesize that enhancement of infection control measures and cautious use of antibiotics may help to control further increase of resistance.

**Electronic supplementary material:**

The online version of this article (10.1186/s13756-019-0522-6) contains supplementary material, which is available to authorized users.

## Background

*Escherichia coli* (*E. coli*) colonize the gut of newborns within hours after birth when the newborn is exposed to the maternal vaginal and fecal flora and to environmental bacteria [1, 2]. Under physiological circumstances *E. coli* living in the colonic mucus and its human host are symbionts [3]. Nevertheless, virulent *E. coli* strains can severely sicken neonates and infants, causing enterocolitis, urinary tract infections, meningitis, and septicemia [4–8].

Increased antibiotic resistance in *E. coli* has been observed in recent years [9–11]; antibiotic-resistant pathogens are generally a growing menace [12–16], especially in low-income countries, where prevalence of resistant bacteria is higher than in high-income countries [17]. In Thailand, rates of antibiotic resistance in *E. coli* are high in healthy adults, food, food animals, and the environment [18].

In literature, there are only few data on the prevalence of antibiotic-resistant bacteria in healthy newborns [19, 20]. Smit and coworkers have found a high rate of neonates (46%, *n* = 17) to be colonized with multidrug-resistant *Klebsiella pneumoniae* within 24 h of admission to a neonatal unit in Cambodia and they have claimed a high within-host diversity [21]. It is well known that antibiotic exposure triggers the development of resistant bacteria [22]. However, it is still a matter of debate how infants acquire resistant bacteria including commensal bacteria of the intestinal microbiota without any antibiotic selective pressure. We sought to investigate the acquisition of antibiotic resistance in *E. coli* from stool of healthy infants in Northern Thailand during the first year of life and to assess whether resistant *E. coli* persist in infant gut as well as whether these microorganisms are transmitted from parent to child. We also investigated which host and environmental factors and which bacterial virulence factors had contributed to frequency and duration of infant gut colonization by antibiotic-resistant *E. coli*.

## Methods

This cohort study was approved, according to the declaration of Helsinki 2000, by the ethics committees of the Medical University of Innsbruck (Ethics approval number: 260/2010) and the Faculty of Medicine, Chiang Mai University (Ethics approval number: 260/2533). Samples were collected between September 2010 and December 2012 at the Department of Pediatrics, Chiang Mai University, and the Meledek (Mother and Child) Hospital, both Chiang Mai, and at the Sanpatong District Hospital, all in Northern Thailand after informed consent of the parents was obtained. Characterizations of the resistant strains were completed in 2017.

### Study participants

All healthy singleton newborns of the participating hospitals that were born during the enrollment period were consecutively invited to take part in the present study. A healthy infant was defined as a newborn with good fetal adaptation (Apgar score at least 9 after 10 min; umbilical cord vein pH > 7.20) without any signs of infection (body temperature < 38.0 °C; C-reactive protein < 0.7 mg/dl or C-reactive protein not measured due to the lack of clinical signs of infection).

Infants with syndromic disorders or malformations influencing the function of the gastrointestinal tract as well as immunodeficiencies, problems of enteral feeding for > 3 days or hospital stays > 14 days were excluded. Infants born to parents with immunodeficiency or malignancy or of mothers given antibiotics during labor were also not enrolled - antibiotic use in infants during the first year of life was no drop out criterion.

The study protocol aimed collection of meconium samples during the first 48 h after birth, infant stool samples at ages 2wk, 2mo, 4 to 6mo, and 1y, and parent stool samples at infant birth. Families in which more than one infant stool sample was missing at the end of the follow-up were withdrawn.

### Investigation of host and environmental factors associated with the development of antibiotic resistance

Demographic and clinical data (gender, birth mode, complications, underlying diseases, antibiotic use, hospital, etc.) and environmental data (siblings, pets, infant feeding) were recorded by caregiving nurses or physicians through personal obtained questionnaires (Table 1, Additional file 1).

**Table 1:** Demographic characteristics of study participants (*n* = 142)

	Number	Proportion (%)
Gender		
Males	75	52.8
Females	67	47.2
Week of gestation		
Preterms (<37wk)	20	14.1
Birth mode		
Vaginal	110	77.5
Caesarean-section	32	22.5
Birth complication of newborn		
Yes	8	5.6
Hospital		
Tertiary urban hospital	90	63.4
Urban birth clinic	31	21.8
Rural hospital	21	14.8
Antibiotic usage during pregnancy		
Yes	30	21.1
Siblings		
Yes	57	42.5
Pets		
Yes	67	47.2
Exclusively breast feeding at		
48 h	95	66.9
2wk	109	76.8
2 m	106	74.6
4-6 m	77	54.2
1y	0	0
Antibiotic usage during 1y		
< 48 h	9	6.3
< 2wk	13	9.2
< 2 m	21	14.7
< 4-6 m	29	20.4
1y	54	38.0
First appearance of AB resistant *E. coli*		
Meconium	49	34.5
< 2wk	89	62.7
< 2 m	113	79.6
< 4-6 m	132	93.0
1y	142	100.0

### *E. coli* collection and characterization

Stool samples were collected by nurses and parents. Parents were trained during hospital stay in collecting a hazelnut-sized portion of freshly voided feces placed in a sterile sample container in a plastic bag, which was kept refrigerated at 3 °C until transport to the laboratory. If culture within 24 h was not practicable, samples were frozen at − 20 °C until further processing (applicable for < 5% of all samples).

Stool samples were plated on a MacConkey agar plate (Becton Dickinson, Cockeysville, MD) and another 4 MacConkey agar plates supplemented with defined concentrations of tetracycline (TET; 4 μg/ml), ampicillin (AM; 8 μg/ml), co-trimoxazole (SXT; 8 μg/ml), or cefazoline (CEF; 8 μg/ml) as described previously [19].

As detection of *E. coli* from stool culture on MacConkey agar plates may suffer from poor sensitivity due to overgrowth, we have decided to include MacConkey agar plates supplemented with antibiotics. We have chosen TET, AM, SXT, CEF as screening antibiotics for two reasons: First, these antibiotics are widely used in human and veterinary medicine and especially in treatment of early childhood infection, thus resistance to these antibiotics are an important issue.

Second, the use of tetracyline is obsolete in children below the age of 8 years due to severe side effects. Thus, detection of resistance in infants reveals interesting insights into the problem of creating a resistance pool due to wide use and its persistence. After growth on agar plates, differently appearing colonies from each agar plate were collected for further analyses. To confirm the hypothesis that colonies with same morphological appearance originate from the same clone, we have picked two identical appearing colonies from each plate. Furthermore, colonies with the same morphological appearance on different agar plates were frozen as well. All isolates were transferred to the Division of Hygiene and Medical Microbiology, Medical University of Innsbruck, where the following analyses were performed. The procedure described above was based on a pilot study performed in 10 families in which 5 similar-appearing colonies from stool samples were genetically indistinguishable by pulsed-field gel electrophoresis **(**PFGE; see below).

For identification, isolates were streaked on ChromID CPS medium (bioMérieux, Marcy-l’Étoile, France) and incubated for 24 h at 37 °C. All red or white colonies were considered *E. coli*. Species identification was confirmed by Matrix Assisted Laser Desorption/Ionization- Time Of Flight (MALDI-TOF) (Bruker Daltonik, Bremen, Germany).

All *E. coli* strains isolated according to the protocol detailed above were tested by disc diffusion for resistance to 16 different antibiotics (AM, ampicillin; AMC, amoxicillin-clavulanic acid; CIP, ciprofloxacin; CN, cefalexin; CPD, cefpodoxime; CRO, ceftriaxone; CXM, cefuroxime; ETP, ertapenem; FM, nitrofurantoin; FOS, fosfomycin; GM, gentamcin; MEC, mecillinam; SXT, co-trimoxazole; TET, tetracycline; TMP, trimethoprim; TZP, piperacillin-tazobactam) on Müller-Hinton agar plates according to EUCAST clinical breakpoints (V06/2016) [23] or, for TET, using CLSI standards [24].

### Genomic characterization by PFGE

PFGE was performed only in *E. coli* strains with the same resistance pattern which were isolated in at least 3 different sample time points (infants) or in at least one parental stool sample and two infant samples (resource limitations precluded testing of all *E. coli* cultured from stool samples). PFGE was performed according to a standardized protocol for molecular subtyping of *E. coli* (PulseNet, CDC). PFGE profiles generated by *Xba*I digestion of chromosomal DNA were analyzed using BioNumerics 3.5 software (Applied Maths, Sint-Martens-Latem, Belgium) and clusters were defined as described [25].

### Persistence and transmission

*E. coli* strains with the same antibiotic resistance profile and PFGE cluster isolated from at least 3 different samples from one infant were defined as “persistent”. In families, *E. coli* strains with the same antibiotic resistance profile and PFGE cluster isolated from at least one parental stool sample and at least 2 different infant samples, “transmission” of the strain from parent to infant was inferred.

### Multilocus sequence typing (MLST) and phylogenetic grouping

All persistent and transmitted strains underwent MLST, as did a control group of 7 strains only found once. Gene amplification and sequencing were performed using primers specified at the *E. coli* MLST web site (http//mlst.warwick.ac.uk/mlst/dbs/Ecoli).

Sequences were analyzed using the software package Ridom SeqSphere 0.9.19; sequence types (ST) were computed automatically [26]. Phylogenetic groups of the strains were determined using Structure 2.3.X software based on the concatenated sequences of the 7 housekeeping genes used for MLST (https://web.stanford.edu/group/pritchardlab/structure.html). MLST was performed at the Institute of Hygiene of the University Hospital Münster.

### Virulence and adhesion gene typing

Using multiplex and single PCR, all persistent and transmitted *E.coli* strains as well as the 7 strains in our control group were investigated as described [27] for genes linked with extra- and intra-intestinal pathogenicity, including those encoding for adhesins, iron acquisition, protectins, toxins or invasins, and other virulence factors (Additional file 1).

### Statistics

A sample size of 150 study participants was calculated to estimate the prevalence with an accuracy of ±8%. We applied the Fisher’s exact statistical test to analyze our contingency tables, valid for small sample sizes. A *p*-value of < 0.05 was considered as statistically significant. Univariate analysis of variables of interest was applied. Associations were calculated by using Epi. Info™ 2.2.2.2 software (CDC, Atlanta) and presented as prevalence ratios (PR) including 95% confidence intervals (95%CI).

## Results

**Fig. 1:**
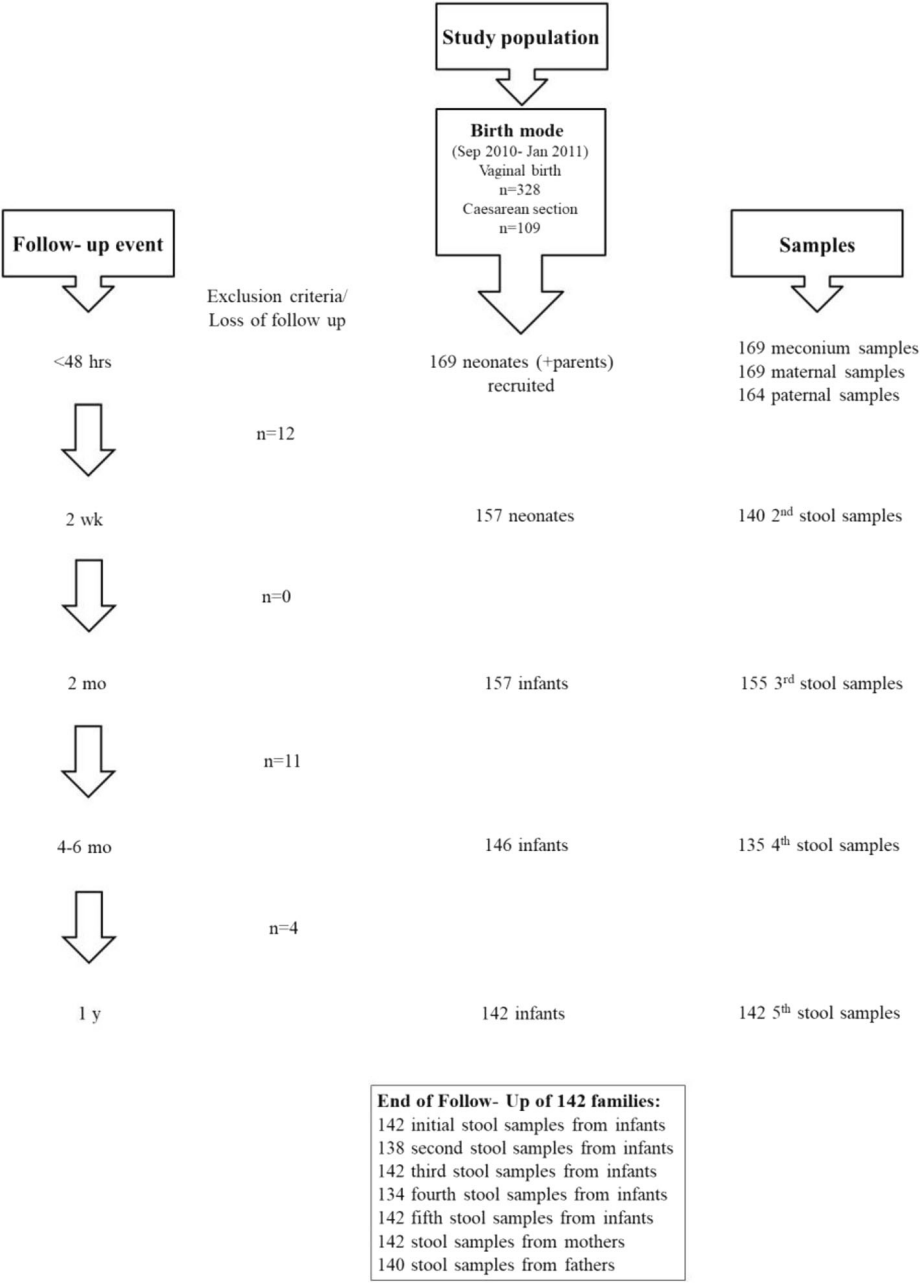
Flowchart of stool sample collection over the one- year study period. Initially 169 families were included. 27 were excluded due to exclusion criteria (> 1 missing stool sample) or loss of follow-up

During the enrollment period 437 babies were born in the participating hospitals (Fig. 1). Among the latter, 169 newborns and their parents fulfilled the inclusion criteria and consented to participate in the study. At the first sampling time point (within 48 h after birth) 169 meconium, 169 maternal and 164 paternal stool samples were collected. During the whole study period 27 families were culled due to lack of more than one stool sample, thus 142 families completed the study (Fig. 1). Among these 142 participants stool samples from 4 infants were not received at 2wk and from other 8 infants at 4 to 6mo.

Ninety of the 142 infants were born at Chiang Mai University Hospital (63,4%), 31 at Chiang Mai Meledek Hospital (urban child birth clinic) (21,8%), and 21 at the rural Sanpatong District Hospital (14,8%); 75 were male (52.8%) and 67 female (47.2%).

All infants were born between week 32 and week 42 of gestation, with 20 (14.1%) before week 37 and 122 (85.9%) were term births. In 103 cases vaginal birth was spontaneous and in 7 it required vacuum support; 32 babies were born by cesarean section (Table 1).

### Prevalence of resistant *E. coli* in parents

One-hundred forty stool samples from fathers and 142 from mothers were included in the study (two fathers were not parental caregivers).

**Fig. 2:**
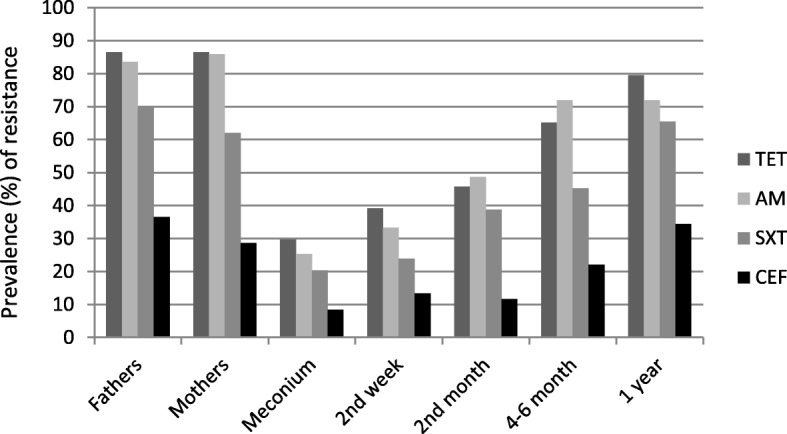
Prevalence of carriage of resistant *E. coli* resistant to tetracycline (TET), ampicillin (AM), co- trimoxazole (SXT), and cefazoline (CEF) in fathers, mothers, meconium of newborns, and infants at 2wk, 2mo, 4 - 6mo, and 1y

In 9 (6%) paternal and 7 (5%) maternal specimens *E. coli* showed no resistance to any of the 4 initially tested antibiotics. Prevalence of carriage of resistant *E. coli* were lowest for CEF (37% fathers, 29% mothers), followed by SXT (70% fathers, 62% mothers) and AM (84% fathers, 86% mothers, Fig. 2), and highest for TET (87% fathers, 87% mothers).

### Prevalence of resistant *E. coli* in meconium and development of resistance during the first year of life

Forty-seven (33%) of the 142 tested meconium samples yielded *E. coli* and all of them showed resistance to at least one of the four tested antibiotics (Table 1). Prevalence of carriage of TET-resistant *E. coli* was 30% (*n* = 42), AM-resistant *E. coli* were found in 25% (*n* = 36), SXT-resistant in 20% (*n* = 29), and CEF-resistant in 8% (*n* = 12).

Prevalence of carriage of resistant *E. coli* steadily increased during the first year of life. For TET, resistance proportions were 39% at 2wk, 46% at 2mo, 65% at 4 to 6mo, and 80% at 1y. The latter almost matched parental resistance proportions. Tendencies were similar for AM, SXT, and CEF (Fig. 2, Table 2).

**Table 2:** Numbers (n) of samples containing resistant *E. coli* and numbers of detected resistant *E. coli* strains in parents and infants during the first year of life

Population	Follow-Up Event	n samples	n samples containing resistant *E. coli* (%)	n detected resistant *E. coli* strains	CN (%)	SXT (%)	AM (%)	FM (%)	TMP (%)	GM (%)	FOT (%)	ETP (%)	AMC (%)	CXM (%)	CPD (%)	MEC (%)	FOX (%)	CIP (%)	TZP (%)	CRO (%)	TET (%)	CEF (%)
Fathers	48 h	140	131 (94)	295	24%	70%	84%	6%	71%	25%	1%	0 (0)	6%	33%	26%	7%	6%	33%	1%	17%	87%	37%
Mothers	48 h	142	135 (95)	263	13%	62%	87%	5%	62%	17%	0 (0)	0 (0)	3%	18%	16%	3%	2%	24%	0%	12%	87%	29%
Infants	48 h	142	47 (33)	65	4%	20%	25%	1%	19%	7%	0 (0)	0 (0)	1%	4%	3%	2%	3%	6%	0%	3%	30%	8%
Infants	2wk	138	70 (51)	71	7%	24%	33%	1%	30%	5%	0 (0)	0 (0)	1%	7%	7%	4%	1%	10%	0%	5%	39%	13%
Infants	2mo	142	84 (59)	99	8%	39%	49%	1%	39%	7%	1%	0 (0)	4%	7%	6%	8%	1%	8%	0%	5%	46%	12%
Infants	4-6mo	134	111 (83)	198	13%	45%	72%	3%	50%	16%	3%	0 (0)	7%	12%	10%	7%	4%	18%	0%	7%	65%	22%
Infants	1y	142	131 (92)	232	18%	66%	72%	1%	65%	27%	1%	0 (0)	8%	17%	18%	9%	2%	27%	3%	13%	80%	35%

### Antibiotic resistance profile of isolated *E. coli* strains

**Fig. 3:**
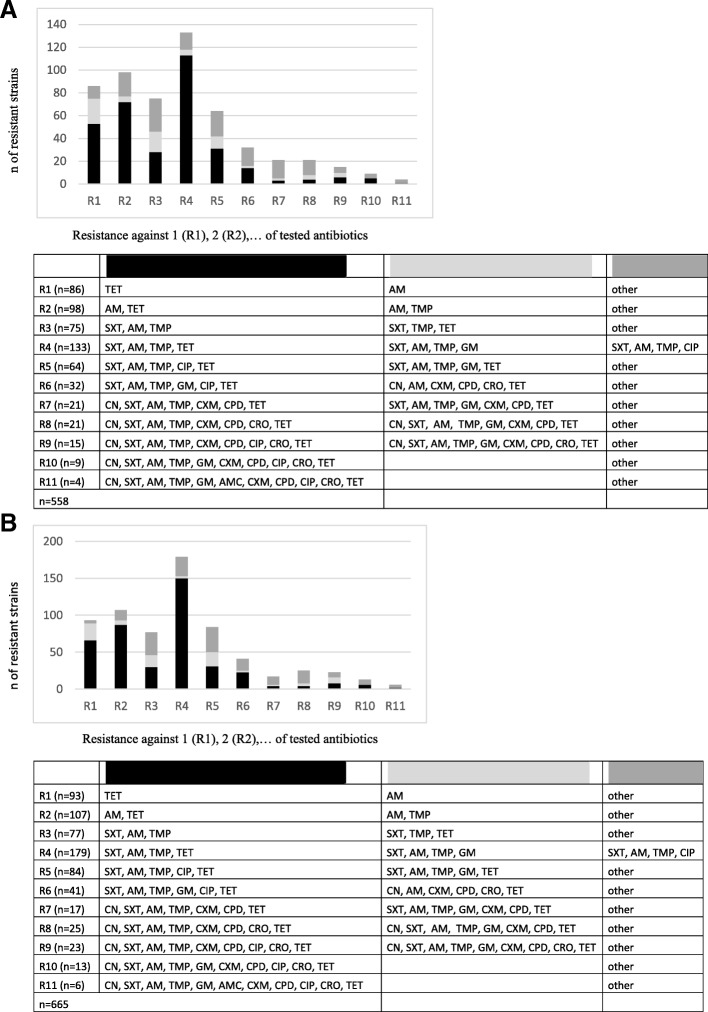
Overview of single-resistance (R1) or multi-resistance (R2-R11) patterns of collected *E. coli* strains in parents (**a**) and infants (**b**). The most prevalent resistance pattern is illustrated in black, the next most in light grey, and others in dark grey. (tested antibiotics: AM, ampicillin; AMC, amoxicillin-clavulanic acid; CIP, ciprofloxacin; CN, cefalexin; CPD, cefpodoxime; CRO, ceftriaxone; CXM, cefuroxime; ETP, ertapenem; FM, nitrofurantoin; FOS, fosfomycin; GM, gentamcin; MEC, mecillinam; SXT, co-trimoxazole; TET, tetracycline; TMP, trimethoprim; TZP, piperacillin-tazobactam)

All resistant *E. coli* strains (*n* = 1223, 558 parental and 665 infant) were tested for multiresistance against 13 additional antibiotics (Fig. 3). The most common resistance pattern in samples was quadruple, viz., resistance to 4 antibiotics (24% in parents, Fig. 3a; 27% in infants, Fig. 3b); most such samples (84%) were resistant to SXT, AM, trimethoprim (TMP), and TET. In 179 strains (86 parental and 93 infantile) single resistance was found, resistance to TET was most prevalent (67%). Multiresistance to 11 different antibiotics was found in 10 samples (1%). None of the tested strains showed resistance to ertapenem. Distribution among children and parents was similar.

Third-generation cephalosporine (ceftriaxone) resistant *E.coli* were found in 4% of meconium samples, 7% at 2wk, 8% at 2mo, 10% 4 to 6mo and 18% at 1y. Prevalence of third-generation cephalosporine resistance after 1y almost reached the prevalence in adult samples (20%) (Table 2).

### Frequency of persistent *E. coli* strains in infants and frequency of transmission of resistant *E. coli* from parents to infants

In 39 families criteria for further PFGE investigation were met (see methods). In 8, the same PFGE clusters were found in parents as well as at least twice in infants (“transmission”); persistence was found in 8 infants. In 4 of the 8 persistent (50%), the same strain was found in one parent (transmission and persistence). Paternal transmission was found in 3 families, maternal transmission in 5.

In most (*n* = 9) of the families meeting criteria for transmission or persistence *E. coli* showed quadruple resistance to SXT, TMP, AM, and TET (75%).

Persistence from meconium sample to half-year stool sample was found in 3 infants, and once each from 48 h to 2mo, from 2wk stool sample to 1y and from 2mo stool sample to 1y.

In 2 infants identical strains were found in meconium sample and in stool samples at 2mo and 4mo but not in 2wk stool sample.

### Association of persistent and transmitted *E. coli* and virulence factors

Molecular examination for various virulence and adhesion factors of *E. coli* strains with PFGE-confirmed persistence or transmission revealed similar distributions of these factors in both transmitted or persistent *E. coli* and non-transmitted and non-persistent *E. coli* (Additional file 1).

### High diversity of *E. coli* strains by MLST

**Fig. 4:**
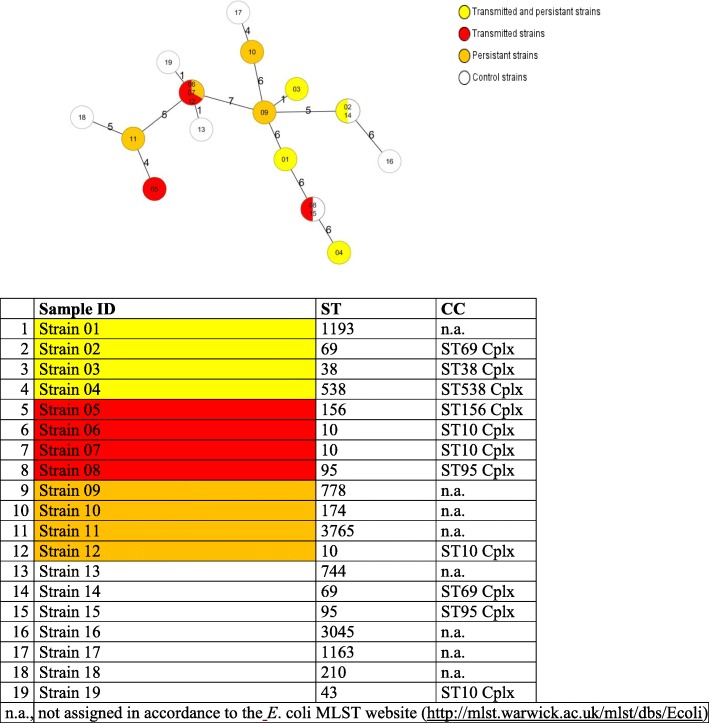
Minimum-spanning tree of the 19 transmitted and persistent (yellow), persistent (orange), transmitted (red), and control strains based on allelic profiles of the respective MLST ST. Each dot represents an allelic profile and the numbers on connecting lines display the number of differing alleles in a pairwise comparison. The dots are named with the isolate IDs and colored according to their assumed epidemiology / origin

MLST revealed a high variety of *E. coli* sequence types among both persistent or transmitted isolates and the control group (Fig. 4). ST10 (phylogenetic group A) was identified 3 times (all persistent or transmitted strains), ST69 and ST95 twice, and ST156, ST38, and ST538 once each. The 9 remaining *E. coli* isolates could not be assigned to any sequence types. Strains with identical STs were found in only 3 families (Fig. 4). Equal numbers of persistent and transmitted *E. coli* strains belonged to phylogroups A, B2, and D (*n* = 4 each). A similar distribution was found in the control group of transient strains.

### Influence of host and environmental factors associated with the development of antibiotic resistance

The difference in prevalence of resistant *E. coli* in infants who received antibiotic therapy during their first year of life and in infants without any antibiotic therapy was assessed using the questionnaire. Altogether 88 infants (62%) did not receive any antibiotics during their first year of life, whereas 54 infants needed antibiotic treatment (Table 1). Antibiotic therapy did not affect the prevalence of resistance. The distribution of resistance patterns was similar in both groups and at the age 1 year of life all infants in our study harbored at least one resistant strain (resistant against at least one of the four antibiotics used for screening) in their gut.

Early prevalence of resistant *E. coli* in meconium was lower in children born by caesarian section (PR: 0.75; CI: 0.60–0.94, p 0.016) compared to vaginally born children. Preterm babies also showed less resistant *E. coli* compared to children born ≥37wk of gestation (PR: 0.73; CI: 0.58–0.92, *p* 0.023). Having been born in the rural hospital was associated with a higher prevalence of resistance (PR: 1.84; CI: 1.06–3.22; p 0.003). Antibiotic usage by the mother during pregnancy showed to be protective (PR: 0.76: CI: 0.61–0.97; p 0.028) (Table 3).

**Table 3:**
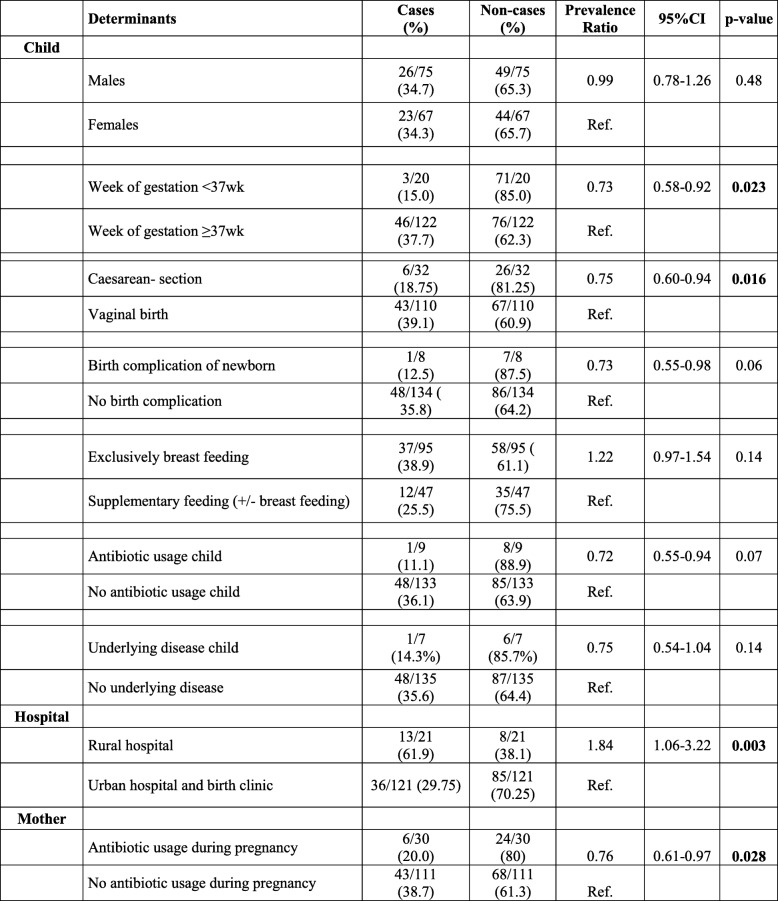
Univariate analysis of determinants associated with prevalence of resistant *E. coli* in meconium

None of the investigated determinants, e.g., birth mode, kind of feeding, exposure to pets, and primary care was associated with persistence or transmission of resistant *E. coli.*

## Discussion

We investigated the colonization of antibiotic-resistant *E. coli* in stool samples of healthy infants in a birth cohort (142 families) over a one-year period.

A culture-based approach was chosen due to several advantages compared to other methods like sequence- based tools especially the possibility to characterize single bacterial species in a stool sample. This method is unique for a big sample size as in the present study and a long observation period of 1 year, however, it might have limitations which are discussed below.

In particular we found high proportions of resistance to TET, AM, and SXT, ranging from 20 to 30%, in *E. coli* from meconium of neonates in northern Thailand. The prevalence of antibiotic-resistant *E. coli* increased during the first year of life, reaching almost the same proportions as in parental samples, with > 70% resistance to TET and AM and 66% to SXT. Resistance against CEF was less frequent, with 8% in meconium samples and 33% in parental stool samples. In 8 children (6%) resistant *E. coli* strains persisted over at least three sampling time points. Early transmission of resistant *E. coli* from parents to children was identified in 8 families (6%). In neither persistent nor transmitted strains an association with any tested virulence and adhesion factor was observed.

The neonatal phase is crucial for intestinal colonization with *E. coli* [1, 28, 29], although this may occur even during the fetal period [30–32].

Of our 142 meconium samples, 47 yielded resistant *E. coli* (33%). Compared with other studies this rate of antibiotic resistance is high: In Innsbruck, Austria, among 21 neonates studied for resistant *E. coli* during the first 48 h of life, resistant *E. coli* (resistant to TET, AM, and SXT) was observed in only one stool sample (4.8%) [19]. AM- resistant *E. coli* were found in 27 of 824 (3.3%) neonatal stool samples in Lithuania [20]. The differences in resistance prevalence may be inferred from differences in study design and geographical background depended on the study location.

Countries belonging to the Organization for Economic Co-operation and Development (OECD) are generally wealthy. In asymptomatic infants antibiotic resistance is much higher in non-OECD countries than in OECD countries: The pooled resistance prevalence in OECD countries to TET was 37.7% (25.9 to 49.7%), to AM, 37.6% (24.9 to 54.3%), and to SXT, 33.5% (2.2 to 71.0%). Resistance in non-OECD countries was higher, with resistance to TET 80.0% (59.7 to 95.3%); to AM, 67.2% (45.8 to 84.9%); and to SXT, 61.4% (no CI given) [17].

Our study confirmed that resistance proportions are high in Thailand, a non-OECD country, with resistance proportions of 80% to TET, 72% to AM, and 66% to SXT in one-year old infants.

One reason for the high prevalence of antibiotic resistance in Thailand in healthy children may lie in wide use of antibiotics, which are available at grocery stores and retail shops without prescription [33]. Moreover, high proportions of antibiotic resistance among bacteria have been found in various environmental sources in Thailand, including farms, water, and food [18]. Additionally, extensive use of antibiotics in livestock worsens the situation by transmission of resistant strains to humans via the food chain [34, 35].

The prevalence of carriage of third-generation cephalosporine-resistant *E. coli* in the present study (both among infants and parents) was rather low compared with other regional investigations [18, 36–39]. Luvsansharav et al. showed local differences in prevalence of ESBL- producing *E. coli* in healthy individuals in different provinces in Thailand varying between 32 and 53.9% [36]. In the present study resistance against the third-generation cephalosporine ceftriaxone was found in only 20%. We did not perform a selective screening for third-generation cephalosporine-resistant *E. coli* using ESBL-chromogenic media or MacConkey agar supplemented with a third-generation cephalosporine. This might have resulted in a low sensitivity.

In the present study the most frequently detected resistance phenotypes were single resistance against tetracycline (TET) (9.7%) and quadruple resistance against AM, TET, TMP, and SXT (21.5%). The high proportion of TET resistance is of interest when considering that TET is contraindicated in children. As persistence of resistant *E. coli* in children and transmission from parents to children was observed in only 6% each, we infer that in our study location, northern Thailand, a reservoir of resistance genes exists, with transmission mainly via horizontal gene transfer. This inference is supported by the finding that different MLST types were found in persistent, transmitted, and control strains. These resistant strains were detected soon after birth and resistance proportions also increased during the first year of life suggesting that parents and the environment are likely to be the main reservoir for these resistance genes. Another hypothesis, which was not investigated in the present study, is that resistant strains have likely been transmitted from the environment or from health-care-workers to the neonate or to the infant. However, exposure time was short when considering that meconium is usually passed within the first 48 h after birth.

Both antibiotic use and day-care center exposure have been described as risk factors for the acquisition of resistant *E. coli* strains in neonates and young children [40, 41]. We found no statistically significant association between antibiotic use and occurrence of resistant *E. coli*.

However, that most children who received antibiotic therapy did so aged between 6mo and 1 yr., and that at 6mo the prevalence of antibiotic resistance was already above 80%, makes it difficult to draw conclusions.

In the present study no data on the attendance of day-care centers were collected. However, attendance of day care centers is common in Thailand and thus it can be assumed that this also had an influence on transmission and colonization with resistant *E.coli* in the present study*.*

Several factors have been proposed as influencing the composition of the intestinal microbiota of infants. In the KOALA Birth Cohort Study of 1032 infants aged 1mo in the Netherlands, mode of delivery, type of infant feeding (breast feeding versus formula feeding), gestational age, and antibiotic use were the most important determinants for development of intestinal flora [42]. In the present study we also found significant lower prevalence of resistant *E. coli* in meconium in children born by caesarian section compared to vaginal delivery. This is consistent with the findings of previous studies showing that delivery mode affects the direct transmission of initial bacteria from mother to newborns [43, 44]. In contrast, birth in the rural hospital was associated with an increased risk of early colonization with resistant *E. coli* strains, antibiotic usage by the mother during pregnancy showed to be protective. We can only speculate about the reasons for this. Less hygienic standards in rural hospitals might be an explanation for this. Some studies showed placental transfer of antibiotics such as erythromycin in humans [45]. This might be a reason for delayed colonization with intestinal resistant bacteria in neonates. However, the reason why maternal antibiotic usage was protective in early colonization with resistant *E. coli* in the present study is not clear and needs further evaluation.

We did not find that feeding behavior [46] or antibiotic usage of the child affected gut colonization with resistant *E. coli*. Other determinants associated with prevalence of resistant *E. coli* in meconium like siblings or pets, were also not identified as significant in our study.

We are aware that our study has several limitations. First, parents were taught by nurses how to collect and to store stool samples and how to keep the samples cooled during transport to the laboratory. Nevertheless, mistakes in sample handling by parents cannot be excluded.

Furthermore, sample collection was initially planned at ages 4mo and 6mo. A flood in northern Thailand in 2011 isolated some study participants for several weeks. The 4mo and 6mo sample data therefore were pooled.

Third, bacterial strains can be excreted intermittently. Thus by taking only one stool sample per time point we cannot exclude to have missed resistant *E. coli*. Furthermore, we cannot exclude that detection of a certain strain was influenced by our sampling processing protocol (including the colony picking) or by any unknown factors*.* Thus, our conclusions on transmission and persistence have to be interpreted with caution. Moreover, antibiotic use in infants during the first year of life may have influenced the diagnostic sensitivity of the culture method.

However, resource limitations precluded testing of all *E. coli* cultured from stool samples.

Another main limitation is the limited number of study participants which decreases the power of our study and thus the ability to detect further determinants associated with development of resistance.

## Conclusion

Antibiotic-resistant *E. coli* was found at high proportions in meconium of healthy Thai neonates (33%) in the present study. This value increased during the first year of life until it almost matched the value among adults. Vertical transmission from parents to their children was detected in only 6% of subjects. We speculate that a high resistance proportion in Thai infants may reflect horizontal gene transfer from reservoirs of resistance genes in parents, healthcare workers, and the environment. We further hypothesize that in order to prevent further increases of resistance, better infection control measures and cautious use of antibiotics are mandatory.

## Additional file

Additional file 1: **Figure S1.** Data collection form **Figure S2.** Genes linked with extra- and intra-intestinal pathogenicity tested by multiplex or single PCR. Comparison between transmitted/persistent E. coli (*n* = 12) and all tested strains (*n* = 20). (DOCX 57 kb)

## Abbreviations

AM: Ampicillin
AMC: Amoxicillin-clavulanic acid
CEF: Cefazoline
CI: Confidence interval
CIP: Ciprofloxacin
CN: Cefalexin
CPD: Cefpodoxime
CRO: Ceftriaxone
CXM: Cefuroxime
*E. coli*: *Escherichia coli*
ESBL: Extended-spectrum beta-lactamase
ETP: Ertapenem
FM: Nitrofurantoin
FOS: Fosfomycin
GM: Gentamcin
h: Hours
MEC: Mecillinam
MLST: Multilocus sequence typing
mo: Months
OECD: Organization for Economic Co-operation and Development
PCR: Polymerase chain reaction
PFGE: Pulsed-field gel electrophoresis
PR: Prevalence ratio
SXT: Co-trimoxazole
TET: Tetracycline
TMP: Trimethoprim
TZP: Piperacillin-tazobactam
wk.: Weeks
