# Supplementary material for: “Enhanced acquisition of antibiotic-resistant intestinal E. coli during the first year of life assessed in a prospective cohort study”
Source: Antimicrob Resist Infect Control. 2019 May 20;8:79. doi: 10.1186/s13756-019-0522-6 (PMC6528363; doi:10.1186/s13756-019-0522-6)
Supplement: Supplementary file 1 — Figure S1. Data collection form Figure S2. Genes linked with extra- and intra-intestinal pathogenicity tested by multiplex or single PCR. Comparison between transmitted/persistent E. coli (n = 12) and all tested strains (n = 20). (DOCX 57 kb) [file 13756_2019_522_MOESM1_ESM.docx]

Supplements


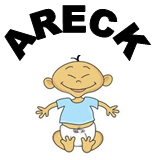


**HN______________________**

**Hospital ____________________________________________________________________________**

**Address ____________________________________________________________________________**

**Phone ____________________________________________________________________________**

1. **Birth and hospital dates of the (<48 hours)**

1.1 Birth mode: ❑ (1) vaginal ❑ (2) cesarean section

❑ (3) other: ………………………………

1.2 Weight (g): ………………………………………

1.3 Length (cm): …………………………………….

1.4 Head circumferenee (cm): ………………………………………

1.5 Week of gestation: ------------------------- (Estimation_________ Ballard for example)

1.6 Apgar score: 1.6.1 ❑ 1 minute ______________ 1.6.2 ❑ 5 minutes _____________

1.7 Childbirth complications:❑ (1) no ❑ (2) yes: (please specify) ______________________

1.8 Does the child have any underlying disease: ❑ (1) no ❑ (2) yes: (please specify) ___________

1.9 Antibiotics received postnatally, infant: ❑ (1) no ❑ (2) yes

| Type(s) of antibiotic(s) | Dose(s) | Duration | Reason(s) |
| --- | --- | --- | --- |
| 1.9.1 |  |  |  |
| 1.9.2 |  |  |  |
| 1.9.3 |  |  |  |

1.10 Length of hospital stay (days): ___________________________________________

1.11 Feeding: ❑ (1) Breast feeding ❑ (2) Formula feeding

❑ (3) Breast and formula feeding ❑(4) Other ________________

**Details, family**

Antibiotics during pregnancy from 6^th^ month until delivery: ❑ (1) no ❑ (2) yes

| Type(s) of antibiotic(s) | Dose(s) | duration | Reason(s) |
| --- | --- | --- | --- |
| 1.12.1 |  |  |  |
| 1.12.2 |  |  |  |
| 1.12.3 |  |  |  |

1.13 Parents live together in one home: ❑ (1) no ❑ (2) yes

1.14 Sisters / brothers: ❑ (1) no ❑ (2) yes

Number of siblings’ ________

1.15 Allergy in family? ❑ (1) no ❑ (2) yes

1.16 Allergic to what? ________________________________________________

1.17 Primary care of the child by: ❑(1) Father ❑(2) Mother ❑(3) Father and mother

❑ (4) Others ______________________________

1.18 Father has underlying disease? ❑ (1) no ❑ (2) yes

Please specify __________________________________

1.19 Mother has underlying disease? ❑ (1) no ❑ (2) yes

Please specify__________________________________

1.20 Pet? ❑ (1) no ❑ (2) yes

1.21 What kind?: ❑ (4) Dog ❑ (5) Cat ❑ (6) Other _______________

**Stool collection data**

1. Stool collected at date.....................

1.1 Resistance:………………………………………………………………………………………

1.2 E.coli strain: ................................................................................................................................

1.3 Klebsiella: ...................................................................................................................................

Filled by.................................date .........../............../............... time............................

**Supplement Figure 1: Data collection form**

| Genes (abb) | *Genes* | Primers | n=12 (100%)  Trans / Pers | n=20 (100%) all |
| --- | --- | --- | --- | --- |
| *afa/draB* | *Afimbrial/Dr antigen-specific adhesin* | s: TAAGGAAGTGAAGGAGCGTG | 2 (17) | 3 (15) |
|  |  | a: CCAGTAACTGTCCGTGACA |  |  |
| *crl* | *Curli fibre gene* | s: TTTCGATTGTCTGGCTGTATG | 12 (100) | 18 (90) |
|  |  | a: CTTCAGATTCAGCGTCGTC |  |  |
| *fimC* | *Type 1 fimbriae (d-mannose-specific adhesin)* | s: GGGTAGAAAATGCCGATGGTG | 11 (92) | 19 (95) |
|  |  | a: CGTCATTTTGGGGGTAAGTGC |  |  |
| *hra* | *Heat-resistant agglutinin* | s: TCACTTGCAGACCAGCGTTTC | 2 (17) | 2 (10) |
|  |  | a: GTAACTCACACTGCTGTCACCT |  |  |
| *iha* | *Iron-regulated-gene-homologue adhesin* | s: TAGTGCGTTGGGTTATCGCTC | 1 (8) | 3 (15) |
|  |  | a: AAGCCAGAGTGGTTATTCGC |  |  |
| *papC* | *Pilus associated with pyelonephritis* | s: TGATATCACGCAGTCAGTAGC | 3 (25) | 5 (25) |
|  |  | a: CCGGCCATATTCACATAAC |  |  |
| *sfa/focCD* | *S fimbriae (sialic acid-specific) and F1C fimbriae* | s: GTCCTGACTCATCTGAAACTGCA | 1 (8) | 1 (5) |
|  |  | a: CGGAGAACTGGGTGCATCTTA |  |  |
| [*tsh a*](file:///C:\Users\Benja\Desktop\GesamtPCR.xlsx#RANGE!tblfn1a) | *Temperature-sensitive haemagglutinin* | s: ACTATTCTCTGCAGGAAGTC | 0 (0) | 0 (0) |
|  |  | a: CTTCCGATGTTCTGAACGT |  |  |
| *mat* | *Meningitis-associated and temperature-regulated fimbriae* | s: TATACGCTGGACTGAGTCGTG | 12 (100) | 19 (95) |
|  |  | a: CAGGTAGCGTCGAACTGTA |  |  |
| *chuA* | *Heme receptor gene (E. coli haem utilisation)* | s: GACGAACCAACGGTCAGGAT | 8 (67) | 13 (65) |
|  |  | a: TGCCGCCAGTACCAAAGACA |  |  |
| *fyuA* | *Ferric yersinia uptake (yersiniabactin receptor)* | s: GCGACGGGAAGCGATGACTTA | 8 (67) | 13 (65) |
|  |  | a: CGCAGTAGGCACGATGTTGTA |  |  |
| *ireA* | *Iron-responsive element (putative catecholate siderophore receptor)* | s: ATTGCCGTGATGTGTTCTGC | 3 (25) | 4 (20) |
|  |  | a: CACGGATCACTTCAATGCGT |  |  |
| [*iroN a*](file:///C:\Users\Benja\Desktop\GesamtPCR.xlsx#RANGE!tblfn1a) | *Catecholate siderophore (salmochelin) receptor* | s: ATCCTCTGGTCGCTAACTG | 3 (25) | 5 (25) |
|  |  | a: CTGCACTGGAAGAACTGTTCT |  |  |
| *irp2* | *Iron-repressible protein (yersiniabactin synthesis)* | s: AAGGATTCGCTGTTACCGGAC | 8 (67) | 13 (65) |
|  |  | a: TCGTCGGGCAGCGTTTCTTCT |  |  |
| [*iucD a*](file:///C:\Users\Benja\Desktop\GesamtPCR.xlsx#RANGE!tblfn1a) | *Aerobactin synthesis* | s: ACAAAAAGTTCTATCGCTTCC | 6 (50) | 12 (60) |
|  |  | a: CCTGATCCAGATGATGCTC |  |  |
| *sitD chr.* | *Salmonella iron transport system gene*  *chromosomally localized* | s: ACTCCCATACACAGGATCTG | 4 (33) | 7 (35) |
|  |  | a: CTGTCTGTGTCCGGAATGA |  |  |
| [*sitD ep. a*](file:///C:\Users\Benja\Desktop\GesamtPCR.xlsx#RANGE!tblfn1a) | *Salmonella iron transport system gene*  *episomally localized* | s: TTGAGAACGACAGCGACTTC | 3 (25) | 5 (25) |
|  |  | a: CTATCGAGCAGGTGAGGA |  |  |
| [*cvi/cva a*](file:///C:\Users\Benja\Desktop\GesamtPCR.xlsx#RANGE!tblfn1a) | *Structural genes of colicin V operon (microcin ColV)* | s: TCCAAGCGGACCCCTTATAG | 2 (17) | 4 (20) |
|  |  | a: CGCAGCATAGTTCCATGCT |  |  |
| [*iss a*](file:///C:\Users\Benja\Desktop\GesamtPCR.xlsx#RANGE!tblfn1a) | *Increased serum survival* | s: ATCACATAGGATTCTGCCG | 2 (17) | 4 (20) |
|  |  | a: CAGCGGAGTATAGATGCCA |  |  |
| *neuC* | *K1 capsular polysaccharide* | s: GGTGGTACATTCCGGGATGTC | 4 (33) | 5 (25) |
|  |  | a: AGGTGAAAAGCCTGGTAGTGTG |  |  |
| *kpsMT II* | *Group II capsule antigens* | s: CAGGTAGCGTCGAACTGTA | 0 (0) | 0 (0) |
|  |  | a: CATCCAGACGATAAGCATGAGCA |  |  |
| *ompA* | *Outer membrane protein* | s: AGCTATCGCGATTGCAGTG | 12 (100) | 20 (100) |
|  |  | a: GGTGTTGCCAGTAACCGG |  |  |
| [*traT a*](file:///C:\Users\Benja\Desktop\GesamtPCR.xlsx#RANGE!tblfn1a) | *Transfer protein* | s: GTGGTGCGATGAGCACAG | 5 (42) | 10 (50) |
|  |  | a: TAGTTCACATCTTCCACCATCG |  |  |
| *astA* | *EAST1 (heat-stable cytotoxin associated with enteroaggregative E. coli)* | s: TGCCATCAACACAGTATATCC | 1 (8) | 1 (5) |
|  |  | a: TAGGATCCTCAGGTCGCGAGTGACGGC |  |  |
| *cnf1/2* | *Cytotoxic necrotising factor* | s: TCGTTATAAAATCAAACAGTG | 0 (0) | 0 (0) |
|  |  | a: CTTTACAATATTGACATGCTG |  |  |
| *sat* | *Secreted autotransporter toxin* | s: TGCTGGCTCTGGAGGAAC | 3 (25) | 6 (30) |
|  |  | a: TTGAACATTCAGAGTACCGGG |  |  |
| *vat* | *Vacuolating autotransporter toxin* | s: TCCTGGGACATAATGGCTAG | 2 (17) | 3 (15) |
|  |  | a: GTGTCAGAACGGAATTGTC |  |  |
| *hlyA* | *Haemolysin A* | s: GTCCATTGCCGATAAGTTT | 0 (0) | 0 (0) |
|  |  | a: AAGTAATTTTTGCCGTGTTTT |  |  |
| *gimB* | *Genetic island associated with newborn meningitis* | s: TCCAGATTGAGCATATCCC | 3 (25) | 4 (20) |
|  |  | a: CCTGTAACATGTTGGCTTCA |  |  |
| *ibeA* | *Invasion of brain endothelium* | s: TGGAACCCGCTCGTAATATAC | 2 (17) | 2 (10) |
|  |  | a: CTGCCTGTTCAAGCATTGCA |  |  |
| *tia* | *Toxigenic invasion locus in ETEC strains* | s: AGCGCTTCCGTCAGGACTT | 2 (17) | 3 (15) |
|  |  | a: ACCAGCATCCAGATAGCGAT |  |  |
| *pic* | *Serin protease autotransporter* | s: ACTGGATCTTAAGGCTCAGG | 0 (0) | 0 (0) |
|  |  | a: TGGAATATCAGGGTGCCACT |  |  |
| *malX* | *Pathogenicity-associated island marker CFT073* | s: GGACATCCTGTTACAGCGCGCA | 9 (75) | 17 (85) |
|  |  | a: TCGCCACCAATCACAGCCGAAC |  |  |

**Supplement Figure 2: Genes linked with extra- and intra-intestinal pathogenicity tested by multiplex or single PCR. Comparison between transmitted/persistent *E. coli* (n=12) and all tested strains (n=20).**
